# Predictors of physical activity at 12 month follow-up after a supervised exercise intervention in postmenopausal women

**DOI:** 10.1186/s12966-015-0219-z

**Published:** 2015-05-05

**Authors:** Fabiola E Aparicio-Ting, Megan Farris, Kerry S Courneya, Ashley Schiller, Christine M Friedenreich

**Affiliations:** Department of Community Health Sciences, Cumming School of Medicine, University of Calgary, 3rd Floor, TRW Building, 3280 Hospital Drive NW Calgary, Alberta, T2N 4Z6 Canada; Department of Cancer Epidemiology and Prevention Research, Cancer Control Alberta, Alberta Health Services, Holy Cross Centre, 2210-2nd Street SW, Calgary, Alberta, T2S 3C3 Canada; Faculty of Physical Education and Recreation, University of Alberta, W1-34 Van Vliet Centre, University of Alberta, Edmonton, Alberta, T6G 2H9 Canada; O’Brien Centre for the Bachelor of Health Sciences, Cumming School of Medicine, University of Calgary, 3300 Hospital Drive NW Calgary, Alberta, T2N 4N1 Canada; Department of Oncology, Cumming School of Medicine, University of Calgary, Tom Baker Cancer Centre, 1331 29th Street NW, Calgary, Alberta, T2N 4N2 Canada

**Keywords:** Recreational physical activity, Adherence, Theory of Planned Behaviour, Randomized control trial, Exercise intervention, Determinants

## Abstract

**Background:**

Few studies have examined recreational physical activity (RPA) after participating in a structured exercise intervention. More specifically, little is known about the long-term effects of exercise interventions in post-menopausal women. This study had two objectives: 1) To compare RPA in postmenopausal women in the exercise group and the control group 12 months after the end of the Alberta Physical Activity and Breast Cancer Prevention (ALPHA) Trial; and 2) To apply the Theory of Planned Behaviour (TPB) to identify predictors of RPA 12 months post-intervention among women in the exercise group.

**Methods:**

Self-reported RPA 12-months post-intervention from a validated questionnaire was used to estimate RPA levels for control group (118/160, 74% response) and exercise group participants (126/160, 79% response). Bivariate analysis was used to compare RPA between exercise and control group participants and to identify TPB variables for multivariate analysis. Logistic regression was applied to TPB data collected from self- administered questionnaires at end of trial by exercise group participants (126/160, 79% response) to identify predictors of long-term RPA.

**Results:**

At 12 months post-intervention, 62% of women in the exercise group were active compared to 58% of controls (p = 0.52). Of the TPB constructs examined, self-efficacy (OR =2.98 (1.08-8.20)) and behavioural beliefs (OR = 1.46 (1.03-2.06)) were identified as predictors of RPA for exercise group participants.

**Conclusions:**

Levels of RPA in the exercise and control groups were comparable 12 months post intervention, indicating that participation in the ALPHA trial was associated with increased physical activity in previously inactive women, regardless of randomization into either the exercise group or in the control group. Exercise interventions that promote self-efficacy and positive behavioural beliefs have the potential to have long-term impacts on physical activity behaviour, although further research is needed to examine additional psychological, social and environmental predictors of long-term RPA in post-menopausal women.

**Trial registration:**

ClinicalTrials.gov NCT00522262.

**Electronic supplementary material:**

The online version of this article (doi:10.1186/s12966-015-0219-z) contains supplementary material, which is available to authorized users.

## Background

Current public health guidelines recommend 150 minutes per week of moderate intensity or 75 minutes per week of vigorous intensity aerobic physical activity for adults in order to obtain health benefits. Substantial evidence supports that being active at recommended levels can reduce the risk of osteoporosis, cardiovascular disease, some types of cancers, diabetes, obesity, high blood pressure, depression, stress, and anxiety [1,2]. However, recent population-level assessments report that only 49% of women between the ages of 55 and 64, and 38% of women over the age of 65 are active in Canada [2].

Considerable evidence exists that inactive but healthy individuals can begin and sustain an exercise program with appropriate supervision and guidance particularly with exercise specialists guiding their uptake of regular exercise [3-6]. There is relatively little research, however, regarding how well these populations can sustain levels of activity without the constant support and encouragement embedded within a structured intervention. Research examining physical activity adherence in general has identified a number of factors that facilitate continued activity in women. Personal characteristics most commonly associated with physical activity behaviour are body mass index, self-rated health status and socioeconomic status [7,8]. Social support has also been identified as a predictor of long-term physical activity in women [9]. Specifically, having friends or family who support participation in regular physical activity, through modelling or as a partner in physical activity, has been associated with long-term adherence [7,10,11]. However, self-efficacy, the self-confidence that one can overcome barriers to be physically active, is the factor most consistently associated with physical activity adherence in women [9,12]. Importantly, as individuals successfully initiate physical activity they gain confidence in their ability to be active, thus encouraging continued participation in physical activity [13].

Few studies have examined predictors specific to long-term regular physical activity after engaging in a structured exercise intervention [6]. These populations offer an excellent opportunity to increase understanding of the patterns and level of adherence to physical activity that exist after participating in a structured exercise intervention. To address this gap in knowledge, the current study had two objectives: 1) To compare recreational physical activity (RPA) in postmenopausal women enrolled in the exercise group and the control group 12 months after the end of the Alberta Physical Activity and Breast Cancer Prevention (ALPHA) Trial; and 2) To apply the Theory of Planned Behaviour (TPB) to identify predictors of RPA 12 months post-intervention among women in the exercise group. This knowledge could inform the design of similar interventions aimed at promoting lasting health benefits.

The Theory of Planned Behaviour (TPB) is a social-cognitive theory often used to understand physical activity behaviour [4-6]. Applied to RPA, the TPB proposes that perceived behavioural control, self-efficacy, instrumental attitude, affective attitude, and subjective norms influence an individual’s intention to engage in RPA which, in turn, is the key predictor of the actual uptake of regular RPA. Perceived behaviour control (a sense of control over the behaviour) is influenced by the perception of factors that may facilitate or impede the behaviour (Control beliefs). Perceived behaviour control and self-efficacy have both been consistently identified as predictors of physical activity engagement and maintenance [4,12,14-16]. Attitude is shaped by behavioural beliefs about the likely consequences of a behaviour and involves the positive or negative perspective towards RPA, including the expected benefits of engaging in RPA (instrumental attitude) and whether or not it is enjoyable (affective attitude). Lastly, subjective norm incorporates the social value of RPA, taking into account other’s opinions and expectations, and how supportive others are of RPA behaviour (injunctive norm).

The ALPHA Trial was a year-long structured exercise intervention developed to examine how aerobic physical activity influences biomarkers hypothesized to be associated with breast cancer risk [17]. The current study examines predictors of continued RPA in the exercise group of the ALPHA Trial 12 months after the intervention was over and regular interactions with the study team ceased. Since adherence to the exercise intervention was high (average 178 (SD = 76) exercise minutes per week over the year-long intervention) [6], we were interested in investigating if these previously sedentary women would continue regular physical activity. Additionally, little is known about the long-term effects on physical activity behaviour of structured exercise interventions, so this study also examines the role of TPB constructs as potential predictors of sufficient RPA to be active according to current physical activity guidelines.

## Methods

### Participants

The study design and methods for the ALPHA Trial have been reported elsewhere [17,18]. Briefly, the ALPHA Trial was a year-long randomized controlled exercise intervention involving postmenopausal women in Calgary and Edmonton, Alberta, Canada. The primary purpose of the trial was to examine the influence of an intensive aerobic physical activity on a number of biomarkers involved in the association between physical activity and breast cancer risk. Women were recruited through either targeted mailings or media campaigns. Eligibility criteria included: age 50–74 years, postmenopausal, no previous cancer diagnosis or major comorbidities, less than 90 min of weekly physical activity, able to do unrestricted physical activity, normal blood lipid and hormone levels, Body Mass Index (BMI weight (kg)/height (m^2^)) between 22 and 40, non-smoker, no medications or exogenous hormone use and not currently or planning to undertake a weight loss program. Three hundred and twenty eligible women were randomized to either a one year exercise intervention (n = 160) or a control group (n = 160). The present study compares RPA of the ALPHA Trial exercise group (n = 126) to that of the control group (n = 118) 12 months post-intervention. The ALPHA Trial protocol, including the 12-month post-intervention follow-up, were approved by the institutional ethics review boards at the former Alberta Cancer Board, the Universities of Calgary and Alberta, and all participants provided written informed consent.

### Exercise intervention

Exercise group participants increased their exercise gradually over the first three months to acix final exercise goal of at least 45 minutes of moderate-to-vigorous intensity aerobic exercise on five days per week (225 minutes/week). At least three sessions per week were supervised by exercise trainers at a recreational facility and up to two sessions per week were unsupervised. The exercise intervention was individualized to the age and fitness level of each woman within these parameters.

Several methods were used to facilitate adherence to the exercise prescription, including regularly scheduled facility-based sessions and telephone follow-up of missed sessions, plans for missed sessions due to vacation or illness, group sessions to encourage social interactions between participants, incentives that were awarded at specific program milestones, regular newsletters and a study website. Adherence was monitored using weekly exercise logs completed by the participants and the trainers.

At the end of the ALPHA Trial, women in the control group were invited to attend an information session, and a one-on-one with an Exercise Trainer to review educational materials explaining how to start and maintain an exercise program. They were also given a free one-month pass for the recreational facility so that they could initiate an exercise regime under the supervision of the exercise trainers.

### Assessment of RPA at 12 month follow-up

Participation in RPA for the 12-month period following the intervention was estimated for exercise group and control group participants through self-report using the Past Year Total Physical Activity Questionnaire (PYTPAQ) [19]. The PYTPAQ assessed all types (occupational, household, and recreational activity) and parameters (frequency, intensity and duration) of physical activity. Since this trial was entirely focused on increasing RPA, only these reported activities were included in this analysis. The intensity of the reported activity was converted to Metabolic Equivalents (MET) values, by assigning a MET value to each reported recreational activity using the Compendium of Physical Activities [20]. Moderate intensity activities were defined as those with a MET value between three and less than six METs and activities with an intensity of six or more METs were vigorous activities. Reported values for frequency and duration for each separate activity were multiplied for a single estimate of the hours per week at moderate or vigorous intensity. Respondents with an average of 150 minutes per week of moderate and vigorous RPA or more over the preceding 12 months were considered to have met physical activity guidelines and categorized as active. Women with less than 150 minutes per week of RPA were considered inactive.

Women were also asked to report the type of activity (walking, cycling, swimming or fitness class), location of activity (home, facility or outdoors) and activity facilitators (gym membership, home exercise equipment, supervised exercise) that they participated in or used in the 12 month period following the end of their participation in the ALPHA Trial.

### Assessment of predictors

#### Demographic and fitness variables

A self-administered baseline questionnaire was used to obtain demographics, medical, reproductive and family history for exercise group participants. Measures of cardiorespiratory fitness and body composition obtained at the time participants completed the ALPHA Trial were used in this analysis. A modified Balke treadmill protocol was used to estimate maximum oxygen consumption (VO_2_ max) from submaximal RPA intensities. Weight and height were measured using a balance beam scale and a stadiometer and were used to calculate BMI. Waist and hip circumferences were measured to the nearest 0.1 cm using a metal tape measure. Whole body dual energy x-ray absorptiometry scans were used to assess total body fat and body fat percentage. Intra-abdominal and subcutaneous fat were measured with a single computed tomography slice at the umbilicus.

#### TPB constructs

Psychosocial characteristics of women in the exercise group were obtained through a self-administered questionnaire at the end of the 12-month intervention. The following TPB constructs were measured: instrumental attitude, affective attitude, behavioural beliefs (advantages), behavioural beliefs (disadvantages), self-efficacy, perceived behavioural control, control beliefs, motivation and injunctive norm. Responses for behavioural beliefs and control beliefs were averaged to generate a summary score for each set of items. Please see Additional file 1 for details regarding the questionnaire items and response options for each TPB construct assessed.

## Statistical analysis

Responses to the TPB questionnaire items by women in the exercise group were collapsed into categories to increase predictive power and for ease of interpretation as follows: for instrumental attitude, perceived behavioural control and injunctive norm the two groups were defined as those individuals who had scores below 7 and 7 or above; for affective attitude, self-efficacy and motivation, participants’ responses were collapsed into three categories where the highest score (7) was coded as 2, the second highest score (6) was coded as 1, and all other scores (<6) were coded as 0. Since the data were highly skewed, it was necessary to distinguish between those who had “extreme” responses in comparison to those with “moderate” and lower responses. Please refer to Additional file 2 for frequency distributions of categorical TPB constructs prior to collapsing. Summary scores for behavioural beliefs and control beliefs were continuous variables.

Bivariate analysis utilized Fisher’s exact test, suitable for analyzing small cell sizes, to compare RPA 12 months post-intervention between women in the exercise group and the control group of the ALPHA Trial and to compare active (at least 150 minutes/week of moderate intensity RPA or 75 minutes/week of vigorous intensity RPA) and inactive women in the exercise group by demographic characteristics (age, location, marital status, education, employment status, ethnicity, and family history of breast cancer), fitness variables (BMI, waist circumference, VO_2_ max, intra-abdominal fat, fat mass, lean muscle mass, and percent body fat), and categorical TPB constructs (attitude, self-efficacy, perceived behavioural control, motivation, and norms). T-tests were used to compare behavioural beliefs scores and control beliefs scores between active and inactive women. Variables with p-values <0.10 in the bivariate analysis were fit into a logistic regression. The level of significance was set at p < 0.10 to minimize Type 2 error so that no potentially predictive variables were excluded prematurely. Logistic regression was used to identify predictors of participation in sufficient RPA to meet public health guidelines among exercise group participants. STATA©10 statistical software was used to conduct all data analyses.

## Results

### Study sample

Adherence to exercise prescription during the ALPHA Trial has been reported in detail elsewhere [6,17]. Briefly, women in the exercise group completed an average of 178 (SD = 76) exercise minutes per week and 71% participated in sufficient RPA to meet current public health guidelines of 150 minutes per week. Of the 320 women randomized, 126 (79%) women from the exercise group and 118 (74%) women from the control group responded to the 12 month follow-up questionnaires. Responders were more likely to be older (61.6 ± 5.7 years compared to 58.9 ± 4.5 years), and have a lower BMI (28.8 ± 4.2 versus 30.2 ± 4.6), less intra-abdominal fat (97.4 ± 55.7 cm^2^ versus 117.2 ± 53.1 cm^2^) and lower percent body fat (43.9% ± 5.2% versus 41.8% ± 5.3%) than non-responders.

### RPA behaviour

There was no statistical difference in the proportion of women who were active 12 months post-intervention between randomized groups (Table 1); 62% of women from the exercise group were active compared to 58% of women from the control group. Women from the exercise group reported participating in a number of physical activities, including walking, bicycling, swimming and aerobics classes, both at home and in a fitness facility. However, active exercise group participants were more likely to report participating in aquacize classes, doing activities outside and having joined a fitness club (Table 2).

**Table 1:** **Comparison of recreational physical activity 12 months post intervention between ALPHA Trial randomized groups, Alberta, Canada**

	**Exercise group (n = 126)**		**Control group (n = 118)**		***P*** **-value** ^**b**^
	**No.**	**%**	**No.**	**%**	
Active^a^	78	62%	68	58%	0.52
Inactive	48	38%	50	42%	

^a^at least 150 minutes/week of moderate intensity recreational activity or 75 minutes/week of vigorous intensity recreational activity.

^b^p-value from Fisher’s exact test.

**Table 2:** **Bivariate associations between type and location of activity performed and recreational activity 12 months post-intervention among ALPHA Trial exercise group participants (n = 126), Alberta, Canada**

**Variables**	**Active** ^**a**^ **(n = 78)**			**Inactive (n = 48)**		***P*** **-value** ^**b**^
	**No.**		**%**	**No.**	**%**	
Type of exercise performed (n = 120)						
Walking						
	Yes	77	100%	40	93%	
	No	0	0%	3	7%	0.08
Bicycling						
	Yes	35	45%	10	23%	
	No	42	55%	33	77%	0.13
Swimming						
	Yes	18	23%	12	28%	
	No	59	77%	31	72%	0.68
Aerobics classes						
	Yes	15	19%	0	0%	
	No	62	81%	43	100%	0.27
Aquacize classes						
	Yes	6	8%	8	19%	
	No	71	92%	35	81%	0.04
Location of Exercise (n = 120)						
Home						
	Yes	49	64%	30	70%	
	No	28	36%	13	30%	0.85
Fitness Centre						
	Yes	52	68%	22	51%	
	No	25	32%	21	49%	0.06
Outside						
	Yes	72	94%	36	84%	
	No	5	6%	7	16%	0.03
Exercise facilitators (n = 120)						
Joined a fitness club						
	Yes	43	56%	13	30%	
	No	34	44%	30	70%	0.03
Purchased home exercise equipment						
	Yes	10	13%	11	26%	
	No	67	87%	32	74%	0.47
Participated in supervised exercise						
	Yes	24	31%	8	18%	
	No	53	69%	35	82%	0.40

^a^at least 150 minutes/week of moderate intensity recreational activity or 75 minutes/week of vigorous intensity recreational activity.

^b^p-value from Fisher’s exact test.

### Predictors of RPA 12 months post-intervention

The analysis to identify predictors of RPA 12 months post-intervention focused exclusively on exercise group participants. Bivariate associations between RPA 12 months post exercise intervention for women in the exercise group and predictor variables are presented in Table 3 (demographics), Table 4 (fitness variables), Table 5 (TPB constructs), and Table 6 (behavioural beliefs and control beliefs). Being active was associated with being married (*P* = 0.04), and an average of 225 minutes/week of exercise during the ALPHA Trial (*P* = 0.02). Of the TPB constructs examined, affective attitude (*P* < 0.01), behavioural beliefs (*P* = 0.01) and self-efficacy (*P* = 0.03) were found to be statistically associated with being active. The behavioural beliefs most strongly endorsed by active women were improved wellbeing (70% versus 45% in inactive women; *P* = 0.02), improved energy levels (61% versus 36%; *P* = 0.02), improved physical strength (61% versus 43%; *P* = 0.03) and stress relief (35% versus 18%; *P* = 0.03). Inactive women were more likely to report exercise during the trial was not beneficial for weight loss than active women (55% and 30%, respectively; *P* = 0.01)

**Table 3:** **Demographic characteristics by recreational activity 12 months post-intervention among ALPHA Trial participants in the exercise group (n = 126), Alberta, Canada**

**Demographics**	**Active** ^**a**^ **(n = 78)**		**Inactive (n = 48)**		
	**No.**	**%**	**No.**	**%**	***P*** **-value** ^**b**^
Age (n = 126)					
50 – 59	31	40%	19	40%	
60 – 69	39	50%	25	52%	
70 – 75	8	10%	4	8%	1.00
Location (n = 126)					
Calgary	34	44%	27	56%	
Edmonton	44	56%	21	44%	0.20
Marital Status (n = 126)					
Married/common-law	62	79%	30	63%	
Single	16	21%	18	37%	0.04
Education (n = 126)					
≤ High school	20	26%	16	33%	
≥ College	58	74%	32	67%	0.42
Employment (n = 118)					
Full time employed	34	46%	27	61%	
Not full time employed	40	54%	17	39%	0.13
Ethnicity (n = 126)					
Caucasian	74	95%	45	94%	
Other	4	5%	3	6%	1.00
Family history of breast cancer (n = 126)					
No	57	73%	40	83%	
Yes	21	27%	8	17%	0.20

^a^at least 150 minutes/week of moderate intensity recreational activity or 75 minutes/week of vigorous intensity recreational activity.

^b^p-value from Fisher’s exact test.

**Table 4:** **Bivariate associations between end-of-study fitness variables and recreational activity 12 months post-intervention among ALPHA Trial exercise group participants, Alberta, Canada**

**Fitness variables**	**Active** ^**a**^ **(n = 78)**		**Inactive (n = 48)**		
	**No.**	**%**	**No.**	**%**	***P*** **-value** ^**b**^
Average exercise time during the ALPHA Trial (mins/week) (n = 126)					
0 - <150	9	12%	14	29%	
150 - <225	37	47%	23	48%	
≥225 - 349	32	41%	11	23%	0.02
Body mass index (kg/m^2^) (n = 114)					
19 - < 25	21	30%	10	22%	
25 - < 30	34	49%	17	39%	
≥30	15	21%	17	39%	0.15
Waist circumference (cm) (n = 123)					
65 - < 85	31	43%	12	27%	
85 – <94	27	38%	17	38%	
>94 – 126	14	19%	16	36%	0.09
VO_2_ (ml/kg/min) (n = 122)					
10 - <27	13	17%	13	28%	
27 - <32	22	29%	14	30%	
>32 – 57	41	54%	19	41%	0.27
Intra-abdominal fat (cm^2^) (n = 126)					
10 - <57	36	46%	16	33%	
57 - <115	25	32%	15	31%	
≥115 – 339	17	22%	17	35%	0.21
Fat mass (kg) (n = 125)					
13 - <25	32	41%	17	36%	
25 - <33	32	41%	16	34%	
≥33 – 56	14	18%	14	30%	0.34
Lean muscle mass (kg) (n = 125)					
29.3 - <39	25	32%	14	30%	
39 - <43	28	36%	14	30%	
≥43 – 59	25	32%	19	40%	0.62
Percent body fat (%)(n = 126)					
25 - <39	31	40%	16	33%	
39 - <43	27	35%	16	33%	
≥43 – 58	20	26%	16	33%	0.64

^a^at least 150 minutes/week of moderate intensity recreational activity or 75 minutes/week of vigorous intensity recreational activity.

^b^p-value from Fisher’s exact test.

**Table 5:** **Bivariate associations between categorical Theory of Planned Behaviour (TPB) constructs and recreational activity 12 months post-intervention among ALPHA Trial exercise group participants, Alberta, Canada**

**TPB constructs** ^**c**^	**Active** ^**a**^ **(n = 78)**		**Inactive (n = 48)**		
	**No.**	**%**	**No.**	**%**	***P*** **-value** ^**b**^
Attitude					
Instrumental (n = 125)					
Less than extremely useful	19	25%	16	33%	
Extremely useful	58	75%	32	67%	0.31
Affective (n = 125)					
Less than quite enjoyable	8	10%	18	38%	
Quite enjoyable	51	66%	25	53%	
Extremely enjoyable	18	23%	4	9%	<0.01
Self-efficacy (n = 124)					
Less than moderately confident	13	17%	18	38%	
Moderately confident	44	58%	24	50%	
Extremely confident	19	25%	6	13%	0.03
Perceived Control (n = 125)					
Less than complete control	33	43%	24	50%	
Complete control	44	57%	24	50%	0.47
Motivation (n = 125)					
Less than quite motivated	9	12%	7	15%	
Quite motivated	38	49%	30	63%	
Extremely motivated	30	39%	11	23%	0.08
Injunctive Norm (n = 124)					
Less than strongly agree	17	22%	14	30%	
Strongly agree	60	78%	33	70%	0.40

^a^at least 150 minutes/week of moderate intensity recreational activity or 75 minutes/week of vigorous intensity recreational activity.

^b^p-value from Fisher’s exact test.

^c^measured at end of study, relating to continuing exercise.

**Table 6:** **Bivariate associations between behavioural beliefs and control beliefs and recreational activity 12 months post-intervention among ALPHA Trial exercise group participants, Alberta, Canada**

**Scores** ^**a**^	**Active** ^**b**^ **(n = 78)**	**Inactive (n = 48)**	***P*** **-value** ^**d**^
	**Mean ± SD** ^**c**^	**Mean ± SD** ^**c**^	
Behavioural beliefs			
Advantages	4.87 ± 1.22	4.21 ± 1.49	0.01
Disadvantages	1.97 ± 0.09	2.20 ± 0.75	0.12
Control Beliefs	2.25 ± 0.94	2.58 ± 0.98	0.06

^a^Average response for related questionnaire items.

^b^at least 150 minutes/week of moderate intensity recreational activity or 75 minutes/week of vigorous intensity recreational activity.

^c^Standard deviation.

^d^p-value from two-sided *t*-test.

Predictors with a *P-*value of 0.10 or less were examined in a multivariate logistic regression model for associations with being active 12 months post-intervention (Table 7). These included marital status, waist circumference, average exercise time during the ALPHA Trial, affective attitude, self-efficacy, motivation, and average scores on behavioural beliefs and control beliefs. Self-efficacy and behavioural beliefs remained statistically significant and positively correlated with being active 12 months post-intervention. Women who were moderately confident that they could continue exercising after the end of the intervention were three times more likely (Odds Ratio (OR) =2.98; 95% confidence interval (CI): 1.08-8.20) to be active at follow-up. Every one unit increase in behavioural beliefs score was associated with 46% (OR = 1.46; 95% CI: 1.03-2.06) increased likelihood of being active 12 months post-intervention. The area under the Receiver-Operator Curve (ROC) for the model was 0.756, indicating that the model has good predictive value.

**Table 7:** **Logistic regression model for predictors of being active** ^**a**^ **12 months post intervention among ALPHA Trial exercise group participants, Alberta Canada**

**Predictor**	**Beta coefficient (95% CI** ^**b**^ **)**	**Odds ratio (95%CI)**
Marital status		
Married/common-law	ref	1.00
Single	-0.82 (-1.77 to 0.13)	0.44 (0.17 to 1.14)
Waist circumference (cm)		
65 - < 85	ref	1.00
85 – <94	-0.57 (-1.68 to 0.54)	0.54 (0.19 to 1.58)
≥94 – 126	-0.94 (-2.12 to 0.23)	0.34 (0.11 to 1.07)
Average exercise time during ALPHA Trial (mins/week)		
0 - <150	ref	1.00
150 - <225	0.40 (-0.83 to 1.64)	1.50 (0.43 to 5.17)
≥225 - 349	0.77 (-0.63 to 2.18)	2.18 (0.53 to 8.86)
Affective attitude		
Less than extremely enjoyable	ref	1.00
Extremely enjoyable	0.52 (-0.91 to 1.94)	1.68 (0.40 to 6.98)
Self-efficacy		
Less than moderately confident	ref	1.00
Moderately confident	1.09 (0.08 to 2.10)	2.98 (1.08 to 8.20)
Extremely confident	1.18 (-0.30 to 2.64)	3.25 (0.73 to 14.00)
Motivation		
Less than extremely motivated	ref	1.00
Extremely motivated	0.12 (-0.96 - 1.21)	1.13 (0.38 – 3.36)
Behavioural beliefs (Advantages)	0.38 (0.03 to 0.72)	1.46 (1.03 to 2.06)
Control beliefs’	0.09 (-0.43 to 0.60)	1.08 (0.65 to 1.83)

^a^at least 150 minutes/week of moderate intensity recreational activity or 75 minutes/week of vigorous intensity recreational activity.

^b^Ninety-five percent confidence interval from maximum likelihood estimation.

## Discussion

Adherence to the exercise intervention in the ALPHA Trial was very good: 71% of women in the exercise group participated in sufficient exercise to meet public health guidelines of 150 minutes per week over the entire year [6]. While only 62% of these women remained active 12 months post intervention, this level of activity is a considerable increase from baseline, at which point all women were inactive (less than 90 minutes/week of exercise). This long-term increase is notable considering that only 43% of Canadian women 45 years and older were considered active in 2008 (Cansim Table 105-0501). ALPHA trial participants, both exercise and control group, were inactive (<90 minutes of moderate to intense physical activity per week) prior to enrollment. The fact that 60% of participants were active at levels recommended for a healthy lifestyle indicated that enrollment in the ALPHA trial was associated with an increase in RPA, regardless of membership to a particular randomized group. Other year-long exercise interventions have also reported increased physical activity in both intervention and control group participants at 12-month follow-up [5].

No difference in levels of RPA between intervention and control groups was observed 12 months post-intervention. The lack of a statistical difference in RPA between exercise group and control group participants may be due to non-response bias. At the 12 month follow-up, non-responders in the control group were more likely to have reported less RPA (mean 11.4 ± 15.1 MET-hours/week) than responders (16.3 ± 16.8 MET-hours/week) at the end of the year-long trial. Since there were more non-responders in the control group, RPA may have been more overestimated in the control, resulting in the observation of a null-effect. Alternatively, many of the women who provided informed consent for the ALPHA trial were interested in becoming active and joined the trial in hopes of being randomized to the intervention group. It is possible that once participation in the ALPHA trial concluded, women in the control group initiated their own exercise routines. The cross-over period at the end of the trial, where women in the control group were offered an educational session and trainer support for initiating an exercise regime, may have encouraged women in the control group to become active.

Our findings are consistent with other intervention studies in post-menopausal women. The Sex Hormones and Physical Exercise Trial (SHAPE Trial) examined exercise maintenance 12 months after a year-long exercise intervention in healthy, postmenopausal women in the Netherlands [5,21]. Similar to the current study, SHAPE Trial investigators reported significantly increased physical activity adherence levels at 12 months post-intervention compared to baseline [5]. Even longer term impacts have been observed. A randomized walking trial intervention in post-menopausal women in the United States found higher levels of physical activity among women randomized into the intervention group than in the control group 10 years after the end of the trial [22]. Furthermore, women in the original walking group were significantly less likely to report heart problems than women in the control group, thus highlighting the potential long-term health benefits of short-term exercise interventions. Participation in the ALPHA Trial improved physical activity behaviour for at least a year after the intervention and the possibility for continued physical activity beyond 12 months in participants exists.

Of the TPB constructs, only self-efficacy and behavioural beliefs related to the perceived benefits of being active predicted long-term RPA in the multivariate analyses. A review by van Stralen et al. [15] concluded that the original TPB constructs are not effective in capturing exercise maintenance and suggested that more emphasis should be placed on enhancing self-efficacy to promote RPA in the general population. There may be several reasons for the lack of predictive support for other TPB constructs, including perceived control, attitudes and normative beliefs in this sample of women. First of all, because of the strict inclusion criteria and participant screening, women who participated may not be representative of the general population from which they were sampled. Instead, they were more educated, health conscious and willing to make and sustain lifestyle changes than other women who were not included. In addition, the study was tightly controlled and efficacy-based since the primary objective of this trial was to examine the effect of exercise on breast cancer biomarkers. Since these participants likely joined the study with high levels of motivation and positive attitudes about exercise, the ability of these constructs to predict exercise was significantly restricted [14]. Moreover, the support provided by the ALPHA Trial Exercise Trainers during the intervention was effective in creating behaviour changes and promoting related self-efficacy without any other motivational factors explained by the TPB [6]. Highlighting the health benefits of physical activity during the intervention could also have enhanced behavioural beliefs, thus improving the possibility of long-term RPA adoption.

The strongest predictor of being active post-ALPHA Trial was self-efficacy, the psychological construct most consistently linked to physical activity behaviour [7]. Exercise self-efficacy was not associated with adherence during the trial [6] but since physical activity was structured during the trial, self-efficacy may not have been as important. However, the ALPHA Trial may have increased exercise self-confidence in participants, especially since two sessions per week were unsupervised and participants were responsible for these sessions. This weekly requirement for home-based sessions gave participants the opportunity to develop confidence through success in adhering to their exercise regime when unsupervised and in a location of their choosing, thus promoting self-efficacy for future engagement in RPA. Notably, the majority of exercise group participants who remained active in the ALPHA Trial joined a recreational facility upon completion of the research study. This type of setting provided the resources and facilities that existed for the ALPHA Trial participants and provided a familiar context within which women may have felt an increased confidence to engage in RPA.

McAuley and colleagues also identified self-efficacy as direct predictor of continued physical activity in older adults 18 months after completing a structured six-month walking intervention [12]. They found exercise frequency and support for exercise during the intervention to promote self-efficacy. It is possible that building a routine of two unsupervised exercise sessions per week and providing Exercise Trainer support during the ALPHA Trial may have increased self-efficacy through a similar mechanism, although further investigation is needed to confirm this hypothesis. Future exercise interventions should consider opportunities for supported unstructured physical activities to build self-efficacy for long-term impact.

Increased self-efficacy may have also played a role in encouraging control participants to increase their participation in RPA after the ALPHA Trial. Although they did not receive the year-long structured exercise intervention, the support, skills and encouragement received during the cross-over period at the end of the trial may have increased control group participants’ confidence to engage in RPA. The free one-month pass for the recreational facility access to supervision by the exercise trainers may have been especially motivational. However, since data regarding who in the control group actively engaged with the resources available, it is difficult to ascertain how much the control group benefited from the cross-over period in terms of long-term RPA.

Notably, responses to the self-efficacy construct were highly skewed towards the positive end of the response scale. While this may be partially a consequence of social desirability bias, the skewness towards favourable responses may also be attributable to the positive effects of the intervention; mainly building up levels of physical activity self-efficacy. However, our findings suggest that women who reported moderate levels of self-efficacy at the end of the Trial were the most likely to be active 12 months post-intervention. Women who reported being extremely confident in their ability to continue being active may have underestimated the obstacles that they would face, while women who were moderately confident might have taken a more guarded approach such that barriers to exercise were expected and they were prepared to overcome these barriers. These findings suggest that exercise interventions aiming for long-term adherence to physical activity should consider additional training for transitioning women from structured programs to an unstructured, self-guided physical activity routine.

Behavioural beliefs have also been found to facilitate long term engagement in exercise among women [9]. Exercise group participants with more positive behavioural beliefs were more likely to be active 12-months post-intervention. Similar to studies in other populations, women in this study reported stress reduction, improved energy levels, well-being, weight loss and increased fitness as important consequences of exercise [23-25]. While some studies suggest that perceived benefits of exercise are not associated with physical activity uptake, others have found that they influence physical activity maintenance [23,24,26]. Many of the long-term benefits of physical activity are not immediately obvious, such as benefits related to reduced illness or improved health [27,28]. However, once some benefits become apparent during the exercise intervention, they may encourage continued participation in physical activity. Coupled with increased self-efficacy through successful adherence to the intervention, behavioural beliefs may have increased the motivation to continue being active among exercise-group participants in this study.

Conversely, control beliefs were not associated with RPA 12 months post-intervention in the ALPHA Trial. Women in the exercise group reported few obstacles to continued exercise; consistent with other studies of older adults, the most common barrier reported were limitations because of medical or health problems [22,29,30]. Participation in the ALPHA Trial exercise group may have provided experience with managing barriers so that by the end of the trial, motivation to engage in physical activity was no longer influenced by perceived barriers. Further research regarding effective strategies for addressing impeding factors specific to post-menopausal women is needed to promote long-term uptake RPA in this population.

Our findings suggest that while some TPB constructs are relevant predictors of long term adherence, it may be useful for future interventions to include a more ecological approach. For example, there is growing evidence for the role of social and physical environmental factors as both direct and indirect facilitators of long-term physical activity maintenance in older adults [12,31]. Social support has been found to increase both adherence within exercise intervention and long-term participation RPA in the older women, potentially by improving perceived behavioural control and increasing self-efficacy [12,16,32]. For women in particular, social support specific to physical activity may increase motivation to be active by legitimizing the time and effort that must be redirected from family and other responsibilities [33]. The built environment has also been identified as an influential factor of physical activity behaviour in women [34]. Since many women report walking as a preferred physical activity, built environments that facilitate walking through safety, comfort, and aesthetics are especially important [35,36]. Recent evidence suggests that psychosocial and built environment factors act synergistically to facilitate physical activity. Social support and neighbourhood walkability, in particular, interact to create an overall positive environment for engaging in physical activity that promotes self-efficacy [31]. Further research using a socio-ecological approach that incorporates psychological, social and environmental dimensions is needed to fully understand long-term RPA adherence in post-menopausal women.

The strengths of the current study include the large sample size, the use of a validated theoretical model to examine predictors of RPA adherence, and the detailed assessments of recreational activity using a previously developed and tested questionnaire [19]. The prospective design also allowed for more conclusive evidence for predictors of RPA in menopausal women post-intervention. Limitations of the study include self-reported measurement of RPA and the selective nature of the study population. The majority of women who participated in the ALPHA Trial were married and highly educated, limiting the generalizability of our findings. Broadening the study population to a more diverse subset or finding similar results in a different study population would be recommended.

## Conclusions

This study contributes to the understanding of predictors of long-term adherence to physical activity following a structured exercise intervention. Our findings are of relevance for behaviour change programs to increase physical activity maintenance on a long-term basis. The majority of the previously inactive postmenopausal women were participating in RPA at levels recommended for a healthy lifestyle post intervention and the strongest predictors of long-term adherence were self-efficacy and behavioural beliefs. Additional research into factors that promote long-term engagement of post-menopausal women in physical activity is needed to direct effective public health investment, and to improve health outcomes ultimately in this growing segment of the population.

## Additional files

Additional file 1: Table S1. Theory of Planned Behavior (TPB) constructs and corresponding questionnaire items, ALPHA Trial, Alberta, Canada. Additional file 2: Table S2. Frequency distribution of responses to categorical Theory of Planned Behaviour (TPB) constructs by recreational activity 12 months post-intervention among ALPHA Trial exercise group participants, Alberta, Canada.

## Abbreviations

95% CI: 95% Confidence intervals
ALPHA: Alberta Physical Activity and Breast Cancer Prevention Trial
BMI: Body mass index
OR: Odds ratio
RPA: Recreational physical activity
TPB: Theory of planned behaviour
