# Supplementary material for: Predictors of physical activity at 12 month follow-up after a supervised exercise intervention in postmenopausal women
Source: Int J Behav Nutr Phys Act. 2015 May 5;12:55. doi: 10.1186/s12966-015-0219-z (PMC4423399; doi:10.1186/s12966-015-0219-z)
Supplement: Additional file 1: Table S1. — Theory of Planned Behavior (TPB) constructs and corresponding questionnaire items, ALPHA Trial, Alberta, Canada. [file 12966_2015_219_MOESM1_ESM.docx]

## Table S1– Theory of Planned Behavior (TPB) constructs and corresponding questionnaire items, ALPHA Trial, Alberta, Canada

| **TPB construct** | **Item** | **Likert response scale** |
| --- | --- | --- |
| Instrumental attitude | Now that the exercise training program is over, I think that continuing to exercise would be… | Extremely useless = 1  Quite useless = 2  Slightly useless = 3  Neutral = 4  Slightly useful = 5  Quite useful = 6  Extremely useful = 7 |
| Affective attitude | Now that the exercise training program is over, I think that continuing to exercise would be… | Extremely unenjoyable = 1  Quite unenjoyable = 2  Slightly unenjoyable = 3  Neutral = 4  Slightly enjoyable = 5  Quite enjoyable = 6  Extremely enjoyable = 7 |
| Behavioral beliefs (advantages) | Having completed the exercise training program, I...  …relieved my stress  …improved my energy level  …increased my physical strength  …improved my well-being  …improved my self-image  …lost weight  …slept more soundly | Not at all = 1  2  Somewhat = 3  4  A fair bit = 5  6  Very much = 7 |
| Behavioral beliefs (disadvantages) | Participating in the exercise training program...”  …took away time that I could have spent on other important things  …made me tired and fatigued  …made me sore  …led to injury  …made me eat more | Not at all = 1  2  Somewhat = 3  4  A fair bit = 5  6  Very much = 7 |
| Self-efficacy | How confident are you that you will exercise at this level over the next six months? | Extremely unconfident = 1  Moderately unconfident = 2  Slightly unconfident = 3  Neutral = 4  Slightly confident = 5  Moderately confident = 6  Extremely confident = 7 |
| Perceived behavioural control | How much control do you feel you would have over continuing to exercise? | Very little = 1  2  3  Moderate = 4  5  6  Complete control = 7 |
| Control beliefs | Barriers to my continuing to exercise would be...  …cost  …getting access to a place to exercise  …not having a trainer anymore  …weather  …lack of motivation  …too busy or too little time  …feeling tired or fatigued  …pain or soreness  …medical or health problems | Not at all = 1  2  Somewhat = 3  4  A fair bit = 5  6  Very much = 7 |
| Motivation | How motivated are you to continue exercising? | Extremely unmotivated = 1  Quite unmotivated = 2  Slightly unmotivated = 3  Neutral = 4  Slightly motivated = 5  Quite motivated = 6  Extremely motivated = 7 |
| Injunctive norm | Most people who are important to me approve of me continuing to exercise. | Strongly disagree = 1  Moderately disagree = 2  Slightly disagree = 3  Neutral = 4  Slightly agree = 5  Moderately agree = 6  Strongly agree = 7 |
