# Supplementary material for: Predictors of physical activity at 12 month follow-up after a supervised exercise intervention in postmenopausal women
Source: Int J Behav Nutr Phys Act. 2015 May 5;12:55. doi: 10.1186/s12966-015-0219-z (PMC4423399; doi:10.1186/s12966-015-0219-z)
Supplement: Additional file 2: Table S2. — Frequency distribution of responses to categorical Theory of Planned Behaviour (TPB) constructs by recreational activity 12 months post-intervention among ALPHA Trial exercise group participants, Alberta, Canada. [file 12966_2015_219_MOESM2_ESM.docx]

## Table S2 – Frequency distribution of responses to categorical Theory of Planned Behaviour (TPB) constructs by recreational activity 12 months post-intervention among ALPHA Trial exercise group participants, Alberta, Canada

| TPB constructs ^c^ | Total  (n=126) | | Active ^a^  (n=78) | | Inactive  (n=48) | |
| --- | --- | --- | --- | --- | --- | --- |
|  | No. | % | No. | % | No. | % |
| Attitude |  |  |  |  |  |  |
| Instrumental (n=125) |  |  |  |  |  |  |
| Extremely useless = 1 | 1 | 1 | 1 | 1 | 0 | 0 |
| Quite useless = 2 | 0 | 0 | 0 | 0 | 0 | 0 |
| Slightly useless = 3 | 0 | 0 | 0 | 0 | 0 | 0 |
| Neutral = 4 | 0 | 0 | 0 | 0 | 0 | 0 |
| Slightly useful = 5 | 1 | 1 | 0 | 0 | 1 | 1 |
| Quite useful = 6 | 33 | 26 | 18 | 14 | 15 | 12 |
| Extremely useful = 7 | 90 | 72 | 58 | 46 | 32 | 26 |
| Affective (n=125) |  |  |  |  |  |  |
| Extremely unenjoyable = 1 | 0 | 0 | 0 | 0 | 0 | 0 |
| Quite unenjoyable = 2 | 2 | 2 | 0 | 0 | 2 | 2 |
| Slightly unenjoyable = 3 | 3 | 2 | 2 | 2 | 1 | 1 |
| Neutral = 4 | 4 | 3 | 0 | 0 | 4 | 3 |
| Slightly enjoyable = 5 | 17 | 14 | 6 | 5 | 11 | 9 |
| Quite enjoyable = 6 | 76 | 61 | 51 | 41 | 25 | 20 |
| Extremely enjoyable = 7 | 22 | 18 | 18 | 14 | 4 | 3 |
| Self-efficacy (n=124) |  |  |  |  |  |  |
| Extremely unconfident = 1 | 5 | 4 | 3 | 2 | 2 | 2 |
| Moderately unconfident = 2 | 2 | 2 | 0 | 0 | 2 | 2 |
| Slightly unconfident = 3 | 2 | 2 | 0 | 0 | 2 | 2 |
| Neutral = 4 | 7 | 6 | 1 | 1 | 6 | 5 |
| Slightly confident = 5 | 15 | 12 | 9 | 7 | 6 | 5 |
| Moderately confident = 6 | 68 | 55 | 44 | 35 | 24 | 19 |
| Extremely confident = 7 | 25 | 20 | 19 | 15 | 6 | 5 |
| Perceived Control (n=125) |  |  |  |  |  |  |
| Very little = 1 | 0 | 0 | 0 | 0 | 0 | 0 |
| 2 | 1 | 1 | 0 | 0 | 1 | 1 |
| 3 | 2 | 2 | 0 | 0 | 2 | 2 |
| Moderate = 4 | 22 | 18 | 11 | 9 | 11 | 9 |
| 5 | 10 | 8 | 8 | 6 | 2 | 2 |
| 6 | 22 | 18 | 14 | 11 | 8 | 6 |
| Complete control = 7 | 68 | 54 | 44 | 35 | 24 | 19 |
| Motivation (n=125) |  |  |  |  |  |  |
| Extremely unmotivated = 1 | 1 | 1 | 1 | 1 | 0 | 0 |
| Quite unmotivated = 2 | 0 | 0 | 0 | 0 | 0 | 0 |
| Slightly unmotivated = 3 | 1 | 1 | 0 | 0 | 1 | 1 |
| Neutral = 4 | 1 | 1 | 0 | 0 | 1 | 1 |
| Slightly motivated = 5 | 13 | 10 | 8 | 6 | 5 | 4 |
| Quite motivated = 6 | 68 | 54 | 38 | 30 | 30 | 24 |
| Extremely motivated = 7 | 42 | 34 | 30 | 24 | 11 | 9 |
| Injunctive Norm (n=124) |  |  |  |  |  |  |
| Strongly disagree = 1 | 0 | 0 | 0 | 0 | 0 | 0 |
| Moderately disagree = 2 | 0 | 0 | 0 | 0 | 0 | 0 |
| Slightly disagree = 3 | 0 | 0 | 0 | 0 | 0 | 0 |
| Neutral = 4 | 2 | 2 | 0 | 0 | 2 | 2 |
| Slightly agree = 5 | 3 | 2 | 2 | 2 | 1 | 1 |
| Moderately agree = 6 | 26 | 21 | 15 | 12 | 11 | 9 |
| Strongly agree = 7 | 93 | 75 | 60 | 48 | 33 | 27 |

^a^ at least 150 minutes/week of moderate intensity recreational activity or 75 minutes/week of vigorous intensity recreational activity

^b^ p-value from Fisher’s exact test

^c^ measured at end of study, relating to continuing exercise
